# Comparing the outcome after double level osteotomies in severe valgus and varus knees

**DOI:** 10.1007/s00402-025-05893-x

**Published:** 2025-05-13

**Authors:** Theresa Sendner, Ilona Schubert, Mirsad Spahic, Benoit Reuter, Mario Perl, Jörg Dickschas

**Affiliations:** 1 Sozialstiftung Bamberg, Bamberg, Germany; 2 Universitätsklinikum Erlangen, Erlangen, Germany

**Keywords:** Genu valgum, Genu varum, Double level osteotomy, DLO, Varization, Valgization

## Abstract

**Introduction:**

Osteotomies have played an important role in joint preservation surgery of the knee joint for many years. A double level osteotomy is performed for severe varus or valgus deformities. There are numerous publications on double level osteotomies for severe varus deformities, whereas there are no publications on valgus deformities. The hypothesis of this study was to compare the clinical outcome after varus DLO with that after valgus DLO.

**Material and methods:**

In this retrospective study, 40 DLOs were followed up in 34 patients. In group one (13 cases, age 45.6 (16–61) years) a varization DLO was performed, in group two (24 cases, age 48.3 (20–61) years) a valgization DLO was performed. The pre- and postoperative clinical scores were recorded: Tegner Activity score, Japanese knee society Score and Lysholm Score. The leg axis and knee joint angles were recorded and compared pre- and postoperatively.

**Results:**

The follow-up period was 24 (6–81) months. The follow-up rate was 73% (27/37). The preoperative leg axis in group one showed an average valgus of 15.9° (9–40°). Group two had an average varus of 12° (8–21°). Postoperatively, the leg axis was 3.4° varus in group one and 0.5° valgus in group two. The mLDFA changed in group one from 83.2° to 90.9°, the MPTA from 95.5° to 87.0°. In group two, the mLDFA changed from 91.9° to 85.9° and the MPTA from 83.3° to 88.3° on average. The JLCA changed in group one from − 3.2 (− 5°–0°) to − 0.5° (− 3–2°) postoperative and in group two from 3.3° (1–8°) to 3.0° (0–6°) postoperative. Tegner score, Lysholm score and Japanese knee Society score all improved significantly in both groups. Patients with a valgus axis have worse clinical scores before surgery than the varus group, but the varus group shows a higher potential for improvement postoperatively. Every patient stated that they would have the operation performed again. Complications were rare, two overcorrections required corrective surgery. Two hinge fractures were treated intraoperatively with additional contralateral plate osteosynthesis.

**Conclusions:**

Patients show very good clinical results after DLO. The improvements in the valgus knees are greater, but starting from a lower preoperative level, probably due to improvements in both the lateral compartment and the patellofemoral compartment. An important finding was that JLCA is normalizing in valgization DLO but not in varization DLO. This needs to be considered in planning a DLO.

## Introduction

Osteotomies around the knee joint in the frontal axis played an important role for several years to slow down pain and progression in monocompartimental osteoarthritis [1]. A varization-osteotomy also plays a role in patellofemoral maltracking due to valgus axis [2]. The introduction of angle stable plates for performing open wedge osteotomies [3], mostly the classical medial open wedge high tibial osteotomy (MOW HTO) was responsible for rising numbers in osteotomies around the knee. In high varus deformities, in former times a MOW HTO was performed with high correction angels, leading to pathological high medial proximal tibial angles (MPTA). So the idea of double-level osteotomy (DLO) was born to avoid the resulting oblique joint line [4].

An oblique joint line is defined as a valgus mLDFA smaller 85° combined with a varus MPTA smaller than 85°, most times resulting in a more or less straight axis.

The osteotomies performed in this procedure was the distal femur closing wedge osteotomy (medial or lateral) and medial osteotomies on the proximal tibia (opening or closing). A pre-operative planning on a long-leg radiograph is mandatory [5].

There are already numerous publications in the literature on DLO for genu varum and the outcome [1, 6] but none on DLO for genu valgum.

The aim of this study is to describe clinical and radiological results of DLO in genu valgum and to compare the results with DLO in genu varum.

The following hypothesis should be investigated with this study:

- The clinical outcome after double-level osteotomy for genu valgum is as good as DLO for genu varum.
- Is there a significant deviation from the normal values of the knee joint angles postoperatively?

## Material and methods

### Patients

In this retrospective study, 40 knees in 34 patients who underwent a double-level osteotomy in the frontal axis in the period from 11/2013 to 07/2023 were included (six patients were operated bilateral).

In group 1 with preoperative genu valgum (13 cases) a DLO varization osteotomy was performed. In group 2 with preoperative genu varum (24 cases) a DLO valgization osteotomy was performed.

Three patients with a preoperative oblique joint line (JLCA 0, femoral valgus axis, tibial varus axis) were excluded from the study as they underwent femoral varization and tibial valgization.

The indications for the osteotomy were as shown in Table 1.

**Table 1:** Indications for osteotomy

Indication	Group 1	Group 2
Osteoarthritis	9	21
Fracture-associated osteoarthritis	2	0
Pain without osteoarthritis	2	2
Luxation	0	1

### Inclusion criteria

1. Preoperative genu varum or genu valgum with pathological femoral and tibial knee joint angles.
2. No oblique joint line in the preoperative whole-leg radiograph.
3. Operation in the period from 11/2013 to 07/2023.
4. Postoperative whole-leg radiograph.

### Clinical investigation

For assessment all patients had a clinical examination focused on the axis of the lower limb.

Clinical investigation was performed preoperatively, 6 weeks postoperatively and after full recovery (at least 6 months after surgery).

### Radiography

Patients who presented with medial or lateral knee pain or patellar dislocation and a visual varus or valgus axis obtained a long-leg radiograph and a knee x-ray in lateral view. If there were indications of a torsional deformity in the clinical examination, a torsion-angle computed tomography was performed. An MRI was also carried out to assess the status of the cartilage and meniscus.

### Preoperative planning

For surgical planning before the operation, all cases were measured and planned with a digital planning module (Trauma-CAD; Brainlab, Munich, Germany) and the planning was safed afterwards to the picture archiving and communication system (PACS).

**Fig. 1:**
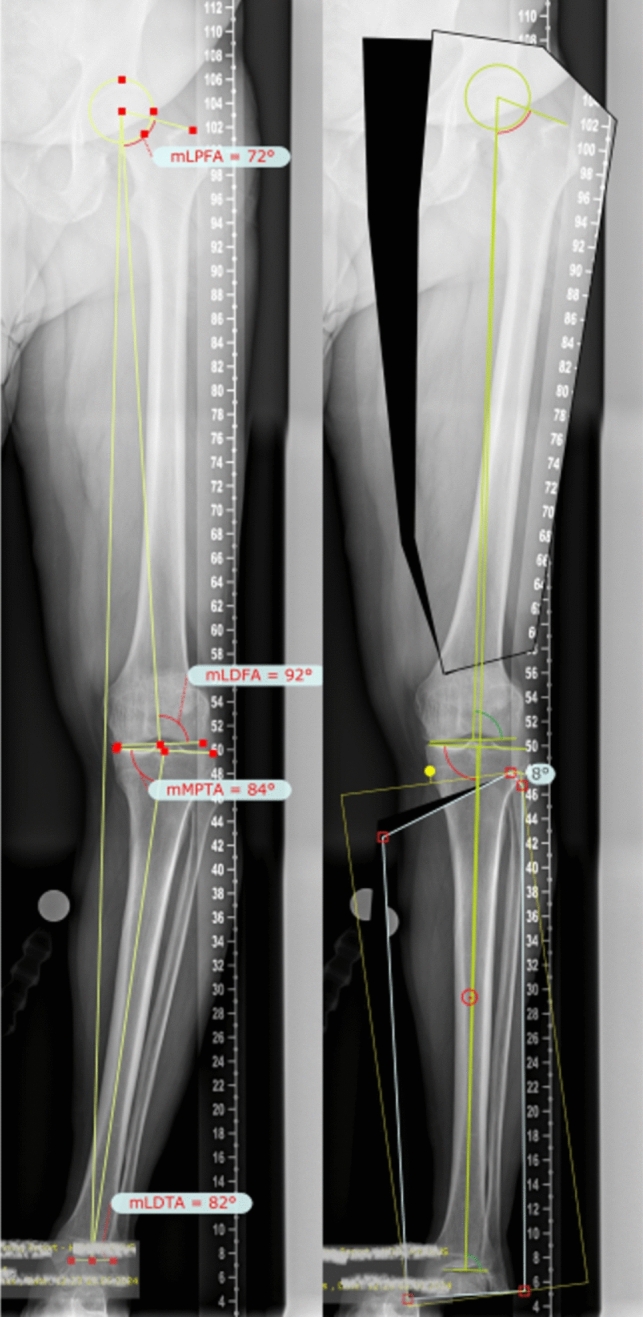
Preoperative long leg view of a patient with severe varus malalignment. The pathological joint angles are measured (left). In the planning software the postoperative result can be simulated and the correction angles can be measured (right)

The knee joint angles were measured in the method after Paley [7] (Fig. 1): the mechanical femorotibial angle (mFTA), mLDFA (mechanical lateral distal femoral angle) and MPTA (medial proximal tibial angle) and the JLCA (joint line convergence angle).

### Surgery

In all patients an arthroscopy was performed before osteotomy to evaluate the status of the cartilage, meniscus and the patellofemoral joint [8]. In 17 cases intraarticular pathologies were adressed for example partial resection meniscus or osteophyte removal, synovectomy, plicar resection or removal of free joint bodies.

For most cases (28/37), an optical navigation system (OrthoPilot Base, Fa. Aesculap) was used for the exact measurements and axis control during surgery. To implement the navigation system the navigation pins were set femoral and tibial, all anatomical landmarks were registered and the femoro-tibial axis was measured.

#### Double level varization osteotomy

**Fig. 2:**
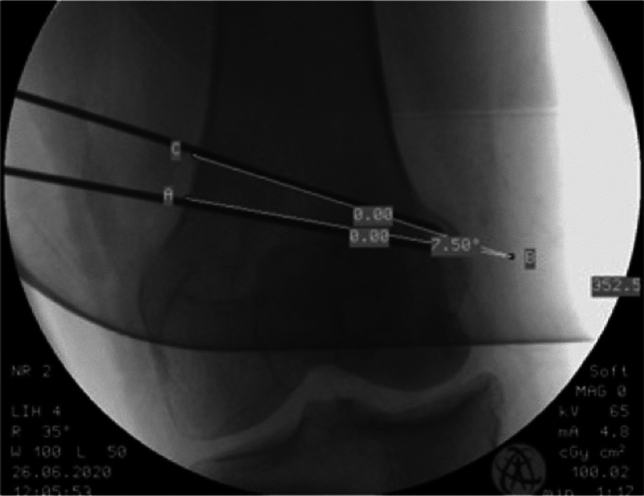
Measuring the angle of the femoral closed wedge osteotomy in the intraoperative fluoroscopy

All DLOs in genu valgum were performed as distal femoral (Fig. 2) and proximal tibial medial closing wedge osteotomies (MCW HTO) in a biplanar technique. Osteosynthesis was performed with angle stable plates. After femoral correction, it is possible to adjust the tibial correction angle compared to the planning depending on the femoral result by using the navigation system.

In MCW HTO a medial collateral ligament release at the tibial insertion is mandatory. After the biplanar osteotomy, the medial collateral ligament should be reefed before positioning the plate. Afterwards fixation with an angle stable plate was done and checked with final x-ray and axis control with the navigation.

#### Double level valgization osteotomy

All DLO in genu varum were treated by lateral distal femoral closing wedge osteotomy and tibial medial open wedge osteotomy except in one case. Here the tibial osteotomy was done from the lateral side as a lateral closing wedge osteotomy because of filling a lateral cyst in the tibial head, a fibular-tibial-arthrodesis and a neurolysis of the peroneal nerve.

For the femoral osteotomy we accessed the femur with a skin incision on the lateral distal femur, the following procedure was nearly the same as in medial closing wedge osteotomy of the distal femur.

**Fig. 3:**
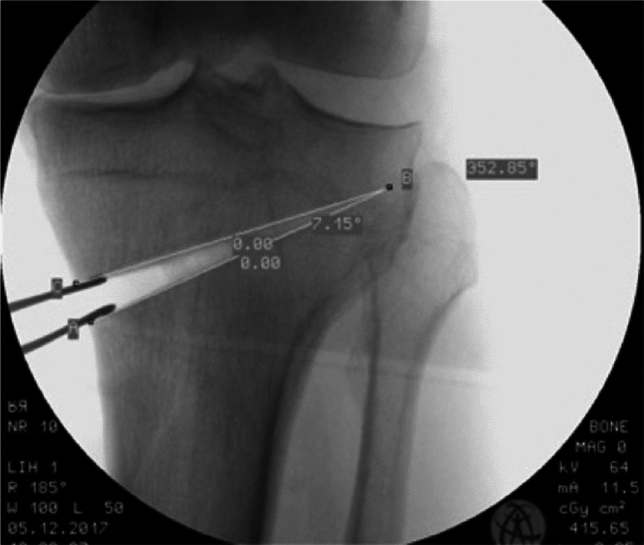
Fluoroscopic measurement of the correction angle in a medial open wedge high tibial osteotomy

Afterwards the medial open wedge HTO was performed with the biplanar osteotomy, the right angle was measured with intraoperative fluoroscopy (Fig. 3). The cancellous bone wedge from the femoral osteotomy is now used in the osteotomy gap as an autogenous cancellous bone graft in some cases. The fixation was done afterwards with an angle-stable plate.

### Post-operative treatment

**Fig. 4:**
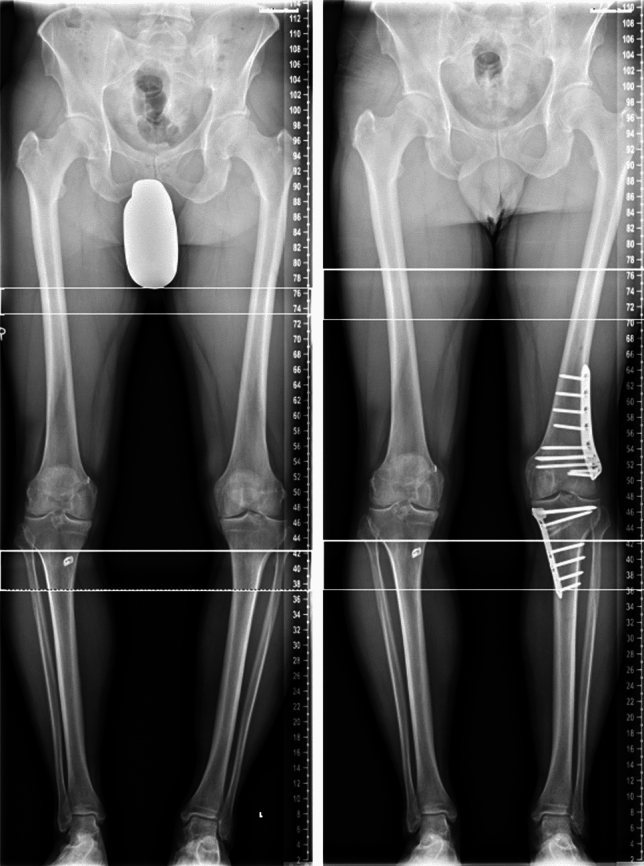
Long leg view of a patient with severe varus axis preoperatively (left) and after double level osteotomy (right)

**Fig. 5:**
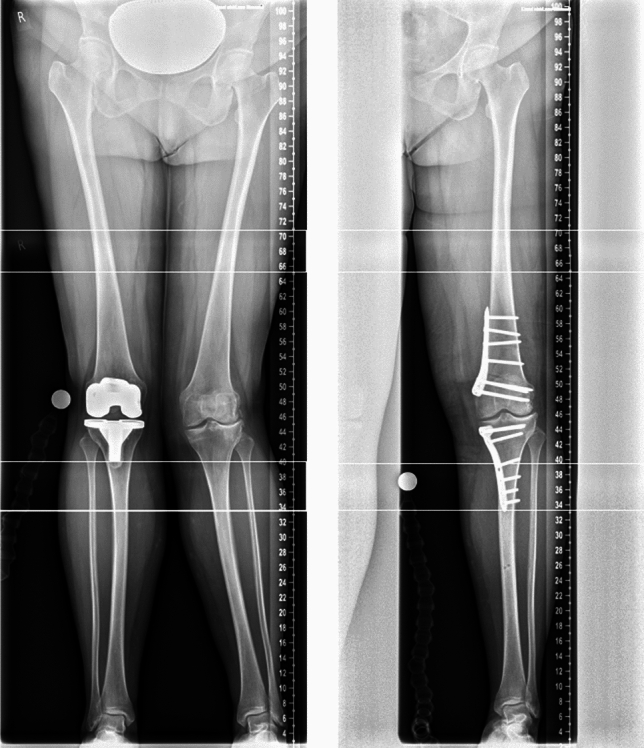
Long leg view of a patient with severe valgus axis before surgery (left, the other knee joint is already replaced by a TKA) and after osteotomy (right)

At the first day after surgery, the mobilization of the knee started with continuous passive motion (CPM) training, additional physiotherapy, lymphatic drainage and cooling. For mobilization forearm crutches with 20 kg weight-bearing were used. Postoperative an X-ray of the knee in lateral and frontal view and if the patient can fully extend also a long-leg radiograph was taken (Figs. 4, 5).

The first appointment in the outpatient department was after 6 weeks with x-ray and clinical examination. Full weight bearing was permitted afterwards.

### Follow-up examination

The follow-up examination consisted of an investigation based on the questionnaire with clinical scores, on the postoperative long-leg radiographs and the clinical examination.

There was only one questionnaire for each patient at the time of the follow-up examination. In this questionnaire, the patient had to use two different colors to mark the function before the operation and at the follow-up time.

### Subjective/objective scores

The following scores were used for assessment pre and postoperative:

- Tegner activity score [9].
- Japanese-knee-society-score [10].
- Lysholm score [11].
- Score from international knee documentation committee (IKDC) [12].
- Question, if they would undergo surgery again.

### Statistical analysis

Statistical analysis was performed with Microsoft Excel and PSPP 2.0.1. The Wilcoxon’s signed rank test was used to determine the magnitude of difference of the intragroup outcome. The Mann–Whitney-U-test was used to determine the differences between the two groups and to prove the probability of a false rejection of the null hypothesis. The probability of a false rejection of the null hypothesis (no group differences) was set at a significance level of p < 0.05.

No funding was received for this study.

## Results

At the time of surgery, the average age in group 1 (varization) was 45.6 (range 16–61) years; in group 2 (valgization) 48.3 (range 20–61) years.

The follow-up period was on average 24 months (range 6–81 months) with a loss-to-follow-up of 27% (10/36). In group 1 the follow-up rate was 61.5% (8/13), in group 2 79.1% (19/24).

2 patients from group 1 (16.6%) had already had a partial lateral meniscus resection before the osteotomy was planned. 6 patients from group 2 (25%) had a partial medial meniscus resection.

### Preoperative and postoperative axis

Preoperative and postoperative axis, knee joint angles and JLCA are shown in Tables 2 and 3.

**Table 2:**
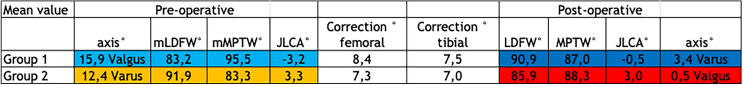
Mean value of radiographic measurements pre- and postoperatively

light blue: Valgus Knees preoperatively, Yellow: Varus knees preoperatively, dark blue: Valgus knee postoperatively, Red: Valgus knee postoperatively

**Table 3:**
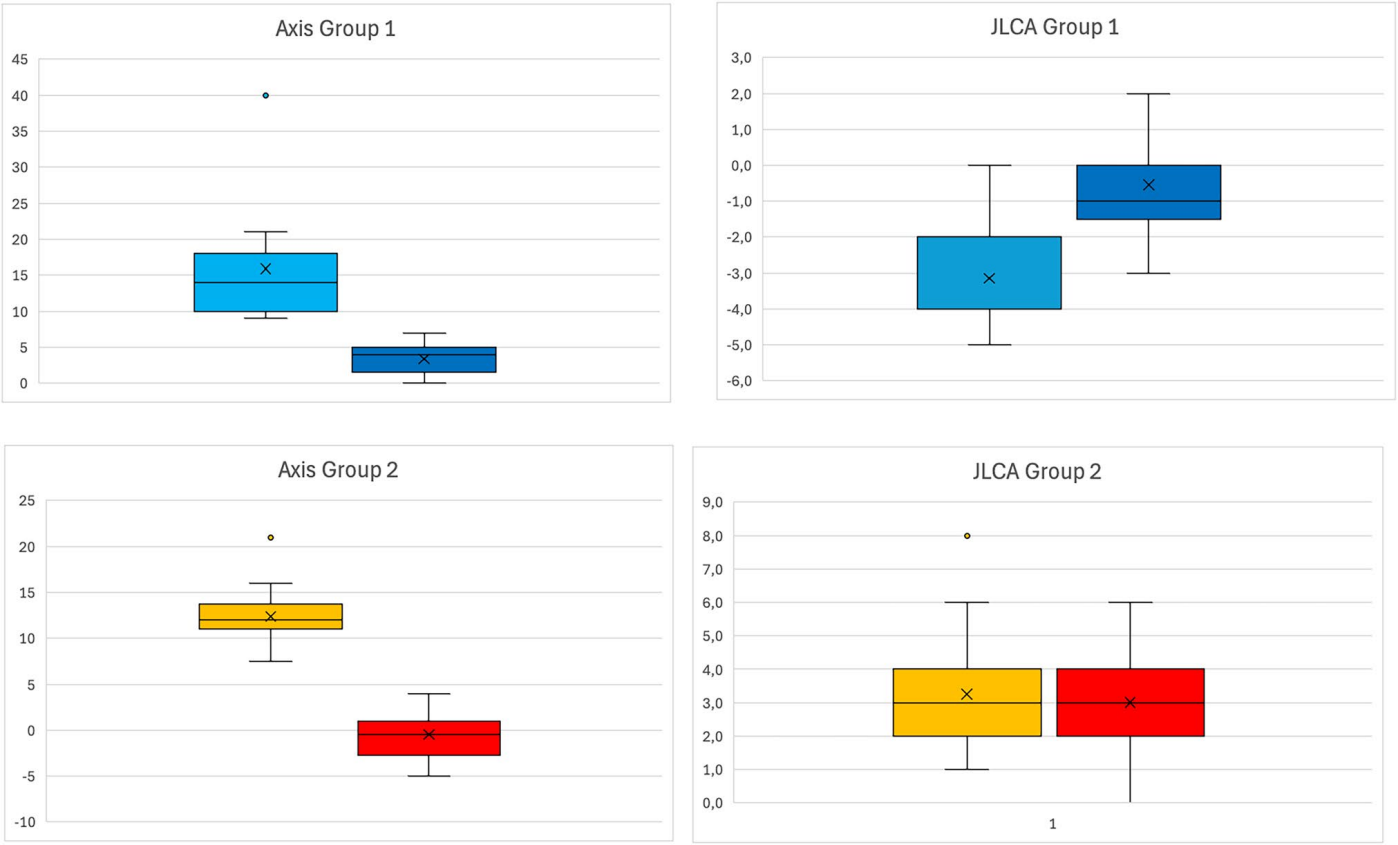
Pre- and postoperative changes in axis an JLCA (Plot)

light blue: Valgus Knees preoperatively, Yellow: Varus knees preoperatively, dark blue: Valgus knee postoperatively, Red: Valgus knee postoperatively

### Results of examination/clinical assessment

Patients with a valgus axis have worse clinical scores before surgery than the varus group, but the varus group shows a higher potential for improvement postoperatively (Tables 4 and 5).

**Table 4:**
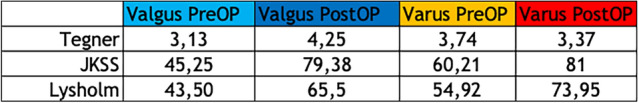
Clinical scores of the varization and valgization group pre- and postoperatively

light blue: Valgus Knees preoperatively, Yellow: Varus knees preoperatively, dark blue: Valgus knee postoperatively, Red: Valgus knee postoperatively

**Table 5:**
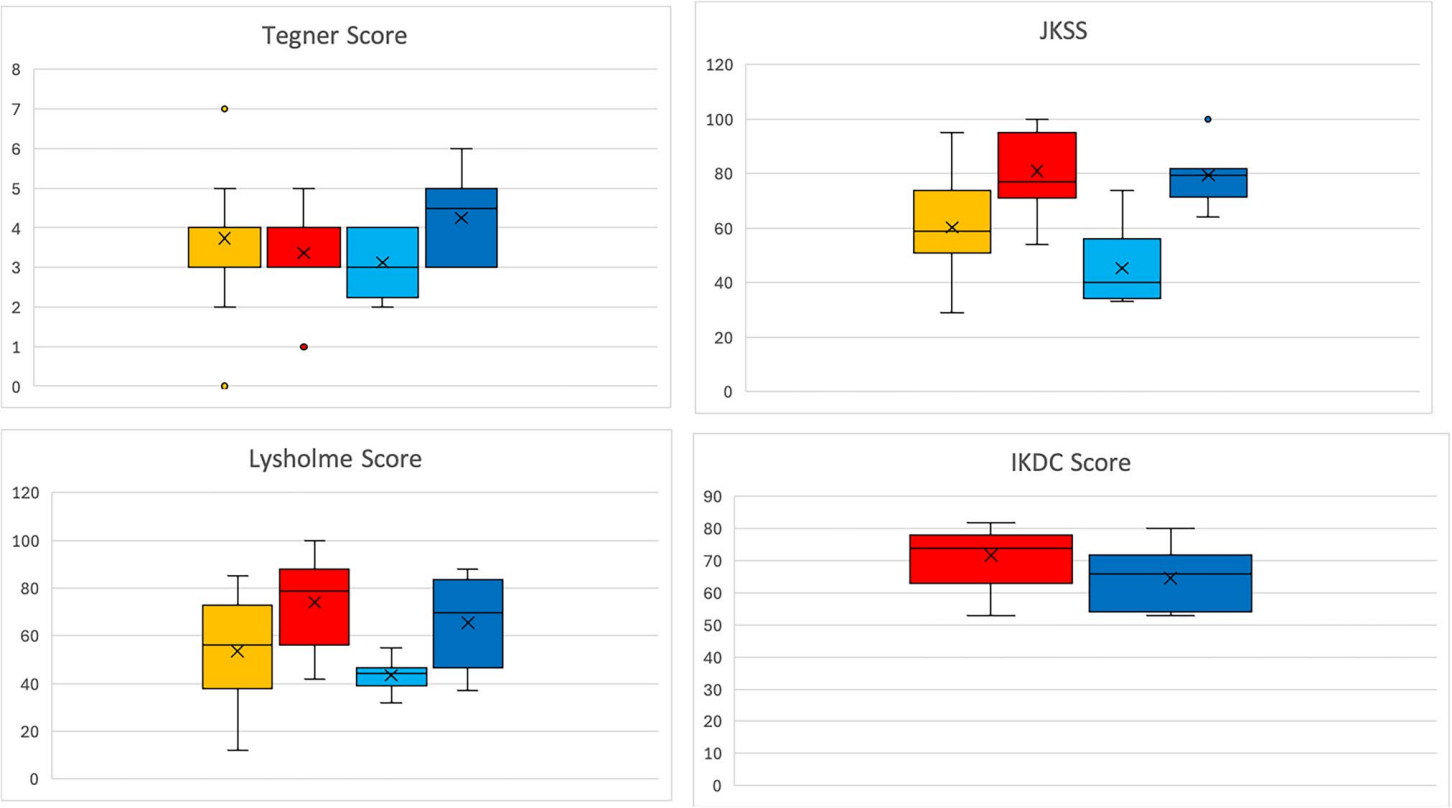
Pre- and postoperative scores (Plot)

light blue: Valgus Knees preoperatively, Yellow: Varus knees preoperatively, dark blue: Valgus knee postoperatively, Red: Valgus knee postoperatively

The postoperative International Knee Documentation Committee score was 64.5 (SD 10, range 53–80) in the valgus group compared to 71.7 (SD 9.1, range 53–82) in the varus group. The final question, if the patient would undergo surgery again was answered from all patients with ‘yes’.

### Complications

In two cases, an overcorrection was detected in the postoperative long-leg radiograph despite intraoperative navigation; leading to an intervention surgery. Two other cases revealed an intraoperative hinge fracture, one tibial and one femoral. These were treated intraoperatively with an additive contralateral plate osteosynthesis. The tibial hinge fracture was in the only lateral closing wedge osteotomy. This osteotomy location was chosen due to lateral tibial head cysts that were filled with bone augmentation.

In none of the cases a pseudarthrosis, infection, deep vein thrombosis or vascular or nerve injury appeared.

## Discussion

This is the first study that compared the results after varization and valgization double level osteotomies. The hypothesis that the clinical outcome after DLO for genu valgum is as good as for genu varum could be proofed. No significant deviation from the normal values of knee joint angles postoperatively appeared. The patients with DLO varization osteotomy achieved a better improvement in clinical scores as the patients with DLO valgization, starting on a preoperatively lower level.

In the early times of medial open wedge osteotomies severe varus deformities were corrected with MOW HTOs with high correction angles, leading to pathological MPTA [13]. This leads to excessive shear stress on articular cartlage [14]. The solution was found in double level osteotomies, dividing the correction on femur and tibia, published by Babis in 2002 [15]. Babis was reporting about 29 DLO with a follow up of 82 month. The results were overwhelming, only one conversion to TKA was reported, and the IKDC score improved from 34 to 64 points. By the way, the idea was not new, Angel was already in 1974 reporting on the results in 67 patients [16]. Even more early in 1969, Benjamin was reporting in British JBJS about double level osteotomy in 57 knees [17]. Several publications were following in recent years, followed by several systematic reviews [18–20]. Alves included in his systematic review 12 studies. But only two studies included showed clinical scores, all others were only reporting about the axis and joint angles. Elbardesy included in his systematic review in 2022 six studies with 175 knees, showing low complication rate and satisfactory short term KOOS and IKDC scores.

The indication for valgization DLO in severe varus knees has to be established when in planning a MOW HTO the MPTA would exceed 92°. Such like limit values do not jet exist for MCW HTO in valgus knees, but we recommend in cases were a MPTA of below 83° would be reached in an isolated HTO to perform a DLO.

While all these publications were concerning about genu varum, publications on double level osteotomies in genu valgum did not appear before 2023. Pioger et al. reported on a case series with 26 patients with severe valgus deformity, performing a DLO using patient-specific cutting guides. The axis and the knee joint angles could be corrected to physiological values. All patients improved in the clinical scores and the average return to sport and work was 4.7 month.

Kuwashima reported on 9 DLO in 8 patients with a valgus deformity in a 25 month follow up [20]. Significant pre- to postoperative improvement was seen for all outcome scores and a high accuracy was reported.

This is the first study comparing the results of Varus and Valgus DLO. In the Valgus group (group 1) of this study, the clinical preoperative clinical scores were lower than in the Varus group (group 2). Our thesis about this fact is that the valgus group did not only suffer from lateral knee pain, but also from patellofemoral problems, including pain. This explains the lower preoperative level, but also the higher improvement after correction, due to the gain of patellofemoral stability with less pressure.

The JLCA was reduced in the varus group significantly from 3.2° to 0.5°, but in the valgus knee it was only reduced from 3.3° to 3.0°. So the normalization of the JLCA only appeared in the varus group. This fact is clinically highly relevant. Why does it not changed in the valgus group? The hypothesis is that severe varus knees regularly showed an extrusion of the most times degenerative damaged medial meniscus, leading to high JLCA. In severe valgus knees an extrusion of the lateral meniscus is regularly not seen, so the lateral meniscus keeps the lateral joint space constant, no matter if a modification in weight bearing of the lateral compartment appears.

Another thesis is that in valgization DLO osteotomies the MOW HTO leads to tightening of the medial collateral ligament (MCL), which prevents the medial compartiment from widening. In the varization DLO a medial femoral and tibial closed wedge osteotomy is performed, so the collateral ligaments are not tightenend so that the lateral compartiment can open more.

Already known increases in the joint-line convergence angle (JLCA) must be considered in the planning to avoid overcorrection. In this study two cases of overcorrection appeared despite intraoperative navigation due to this issue. The recommendation of this study would be that in valgization DLO you must calculate about 2°–3° valgization only by the normalization of the JLCA, in varization DLO this does not have to be calculated.

The study showed some certain limitations. The study group was rather small and there are more varus than valgus knees in the cohort.

The loss-to follow-up was very high with 27% and with the retrospective study design without a control group, the evidence of the study is low.

The recall bias must be viewed critically, as dissatisfied patients would probably not complete the questionnaire. In addition, the follow-up time varied greatly, with the shortest follow-up after 6 months and the longest after 6.7 years.

It can therefore be assumed that the subjective results of the scores change during follow-up treatment, e.g. some patients report a further improvement, especially in everyday life after hardware removal or after surgical treatment of the opposite side.

## Conclusion

Overall, patients who have had a double-level osteotomy are very satisfied. Every patient would undergo surgery again. The axis could be corrected to neutral in both groups, with a slight overcorrection in the varization group.

Patients who receive a bifocal varization osteotomy with a pronounced valgus axis have a lower preoperative level in their clinical scores and showed a higher potential for improvement. This is probably due to the fact that the lateral compartment and also the patellar tracking suffered preoperatively from the severe valgus axis and consequently at the follow up showed a higher improvement by the axis correction.

An important finding was that JLCA is normalizing in valgization DLO but not in varization DLO. This needs to be considered in planning a DLO.

## Data Availability

No datasets were generated or analysed during the current study.
